# Gastrointestinal Parasites, Ectoparasites, and Fungi in Rabbits Attending Clinical Consultations and from Private Owners and Breeders in Portugal

**DOI:** 10.3390/microorganisms13092146

**Published:** 2025-09-13

**Authors:** Carolina Vale, João Lozano, Ana Reisinho, Mariana Louro, Manuela Oliveira, Eva Cunha, Patrícia Lopes, Lídia Gomes, Luís Madeira de Carvalho

**Affiliations:** 1 CIISA—Centre for Interdisciplinary Research in Animal Health, Faculty of Veterinary Medicine, University of Lisbon, 1300-477 Lisbon, Portugal; carolina-vale@edu.ulisboa.pt (C.V.); jlozano@fmv.ulisboa.pt (J.L.); anareisinho@fmv.ulisboa.pt (A.R.); moliveira@fmv.ulisboa.pt (M.O.); evacunha@fmv.ulisboa.pt (E.C.); lidia@fmv.ulisboa.pt (L.G.); 2 Associate Laboratory for Animal and Veterinary Sciences (AL4AnimalS), 1300-477 Lisbon, Portugal; 3 Laboratório de Parasitologia, Instituto Nacional de Investigação Agrária e Veterinária, 2780-157 Oeiras, Portugal; 4 Centre for Ecology, Evolution and Environmental Changes (cE3C) & CHANGE—Global Change and Sustainability Institute, Faculty of Sciences, University of Lisbon, 1749-016 Lisbon, Portugal; patriciaplopes@edu.ulisboa.pt

**Keywords:** rabbits, gastrointestinal parasites, ectoparasites, environmental fungi, Portugal

## Abstract

Few studies have investigated gastrointestinal (GI) and external parasites, as well as environmental fungi, in rabbits using a One Health approach. Between September 2023—May 2024, fecal, hair and skin scraping samples were collected from 72 rabbits that attended clinical consultations and from private owners and breeders in Portugal. Diagnostic techniques included Mini-FLOTAC, direct immunofluorescence antibody, and the analysis of the virulence profile of fur fungi. A total of 58% of the rabbits were positive for GI parasites, namely *Eimeria* spp. (45%), *Cryptosporidium* spp. (32%), *Trichostrongylus retortaeformis* (17%), *Passalurus ambiguus* (13%), *Graphidium strigosum* (13%), and *Giardia* spp. (9%), with only 12% of the infected animals showing clinical signs (diarrhea). In addition, 10% of the animals were positive for *Cheyletiella* sp. infestations. Environmental fungi of the genera *Penicillium*, *Rhizopus*, and *Scopulariopsis* were isolated from 7% of these animals, with the *Scopulariopsis* sp. isolate S1 testing positive for proteinase, lecithinase, and gelatinase activities. Frequent sanitization and regular deworming emerged as essential factors to minimize parasitic frequency. This integrated diagnosis procedure proved to be effective in the search for parasitic and fungal agents in rabbit medicine. Further research is needed to improve the knowledge on the transmission and pathogenicity of these agents in rabbits.

## 1. Introduction

All known breeds of domestic rabbits have a common origin in a single species, *Oryctolagus cuniculus*, which belongs to the family Leporidae, a taxonomic group that includes both rabbits and hares (order Lagomorpha) [1]. To promote the health and welfare of these animals, it is essential to provide proper sanitary management to avoid exposure to several pathogens, namely gastrointestinal (GI) parasites, ectoparasites, and fungi [2].

GI parasites like coccidia of the genus *Eimeria* can lead to clinical disease in rabbits and, in more severe cases, to death. A total of eleven species of the genus *Eimeria* have already been reported in rabbits worldwide, with *Eimeria flavescens* and *Eimeria intestinalis* having the highest pathogenic potential [3]. In Portugal, recent screening studies showed low *Eimeria* spp. prevalences (12–13%) in domestic rabbits [2,3].

Moreover, roundworms of the species *Passalurus ambiguus, Obeliscoides cuniculi*, *Graphidium strigosum*, and *Trichostrongylus retortaeformis* are the most frequent ones in wild rabbits, although they are rarely found in domestic rabbits [4]. A recent study on pet rabbits in Portugal found a low *P. ambiguus* prevalence (3%), which was the only parasite identified [5].

Rabbits are definitive hosts of *Cryptosporidium* spp. and often facilitate its transmission to accidental hosts like humans [6]. A global systematic review regarding *Cryptosporidium* spp. prevalence in 6093 rabbits belonging to nine countries (Australia, Brazil, China, Egypt, Iraq, Japan, Nigeria, Spain, and Tunisia) revealed a *Cryptosporidium* spp. prevalence of 9% and identified three zoonotic species: *Cryptosporidium cuniculus*, *Cryptosporidium parvum*, and *Cryptosporidium andersoni* [7]. In addition, a previous study analyzed the prevalence of zoonotic agents in 438 and 111 fecal samples from rabbits and hares, respectively, in southern Spain and concluded that *Giardia duodenalis* was the most prevalent species (29%), followed by *Cryptosporidium* spp. (1%) [8]. A previous report of a *C. cuniculus* outbreak in humans in the United Kingdom emphasized the need for evaluating the presence of these parasites in pet rabbits under the scope of the “One Health” concept [9].

Furthermore, external parasites can be also harmful to rabbits’ health and to their owners due to the zoonotic potential of some mite species, like *Cheyletiella parasitovorax*, *Notoedres cati*, and *Sarcoptes scabiei* [4]. A previous study performed in rabbits that attended the Veterinary Teaching Hospital of the Faculty of Veterinary Medicine, University of Lisbon (HEV-FMV ULisboa), identified an ectoparasitism frequency of 21%, which was caused mainly by *Leporacarus gibbus*, *C. parasitovorax*, and *Psoroptes cuniculi* [5].

There is a strong correlation between rabbits’ environment and their health status. When provided with specific environmental conditions, such as a relative humidity above 70% and/or temperatures >25 °C, several microbial agents, like fungi, find optimal conditions for their growth and transmission. A study performed in Lisbon and Barcelona, which assessed skin samples from 118 rabbits and guinea pigs, reported the isolation of 11 fungal genera (*Alternaria*, *Aspergillus*, *Chaetomium*, *Cladosporium*, *Mucor*, *Penicillium*, *Phoma*, *Rhizopus*, *Scopulariopsis, Candida,* and *Rhodotorula*), which can potentially lead to skin disease in healthy rabbits and humans [10]. Thus, it is essential to consider environmental fungi in the differential diagnosis of skin disorders, as well their potential zoonotic risk.

The main objectives of this study were to (i) assess the presence of GI parasites, ectoparasites, and fungi in pet rabbits; and (ii) perform an association analysis concerning parasitological and mycological results and several biotic and abiotic factors (animal origin, age, outdoor access, district of residence, housing hygiene frequency, type of household, deworming status, and health status), following a One Health approach.

## 2. Materials and Methods

### 2.1. Studied Rabbits Population

A total of 72 rabbits were sampled for this study, including 29 animals presented for consultation at HEV-FMV-ULisboa and 43 rabbits from external sources (private owners and breeders) located in Portugal (mainland). Between September 2024–May 2025, 69 fecal samples, 72 skin scrapings, and 8 fur samples were collected from these animals. Fecal samples were used to check for the presence of GI parasites, while skin scrapings and fur samples were used for isolation and identification of ectoparasites and fungi.

All animals were handled according to the rules established by the European Parliament and the Council of the European Union (Directive 2010/63/EC, 22 September 2010) [11], and Portugal (DL 113/2013, 7 August 2013) legislation [12]. Samples were collected during routine clinical procedures and always with previous written consent of the owners (Supplementary File S1), and thus, ethical approval was waived for this study. This research study followed a convenience sampling strategy, having been advertised to rabbit owners, who either contacted the authors directly or were contacted by our team, based on filling the criteria of housing a rabbit. Therefore, participation was established based on availability and owner response and not by random selection.

### 2.2. Sampling Procedures

Fecal samples were collected directly from the transport carrier or collected and delivered by the owners. In all scenarios, samples were immediately transported to the Laboratory of Parasitology and Parasitic Diseases (LPDP) of the Centre for Interdisciplinary Research in Animal Health (CIISA-FMV-ULisboa) and stored at 4 °C for a maximum of seven days until further examination.

Moreover, the trichogram and adhesive tape techniques were used to collect samples for detection of ectoparasites [13]. Also, the trichogram technique was used to collect fur samples for isolation of external fungi. These samples were obtained from the edge of active skin lesions on rabbits showing these clinical signs and then stored in a refrigerator at 4 °C for a maximum of 7 days until further processing [14].

### 2.3. Epidemiological Questionnaires

For each rabbit, pre-validated epidemiological questionnaires with a multiple-choice format were prepared and provided to rabbits’ owners to collect data about the animals and their environment (household location, presence or absence of immunocompromised individuals in the household, namely children, elderly, or ill individuals, and age of the animals, exterior access, and frequency of housing hygiene) (Supplementary File S1) [15].

### 2.4. Diagnosis Methods

#### 2.4.1. GI Parasites

To study the presence of GI parasites, different laboratory techniques were performed, namely the Mini-FLOTAC, Willis–flotation, and natural sedimentation techniques. For this purpose, the “3-in-1” technique proposed by Lozano et al. (2021) [16] for exotic animals, which has been extensively performed in routine parasitological diagnosis at LPDP, was applied. This approach consists of the sequential application of three techniques—Mini-FLOTAC, Willis–flotation, and natural sedimentation—using the same sub-sample as opposed to performing them separately. Briefly, for each sample, the fecal suspension contained inside the Fill-FLOTAC device was used to perform not only the Mini-FLOTAC method but also two other techniques, namely, Willis–flotation and natural sedimentation. The implementation of the Mini-FLOTAC technique aimed to morphologically identify nematode eggs and coccidia oocysts based on the descriptions from Thienpont et al. (2003) [17] and Zajac and Conboy (2012) [18] and to quantify their shedding (eggs or oocysts per gram of feces, EPG or OPG, respectively), whereas the Willis–flotation technique was used to search for coccidia oocysts and cestode and nematode eggs, and the natural sedimentation technique was used for the detection of trematode eggs.

The Modified Ziehl–Neelsen (MZN) and the direct immunofluorescence antibody (DFA) techniques aimed to assess the presence of *Cryptosporidium* spp. oocysts and *Giardia* spp. cysts in rabbit fecal samples. For this purpose, samples were first subjected to the concentration method described by Louro et al. [19]. For both methods, the identification of *Cryptosporidium* spp. oocysts and *Giardia* spp. cysts followed the procedures and descriptions from Casemore et al. and Louro et al. [19,20], with *Cryptosporidium* spp. oocysts being nearly round, at 2–6 μm in diameter, and with *Giardia* spp. cysts having an oval format, at 8–12 μm length. In addition, these parasitic forms were stained with fuchsin in the MZN method, in opposition to the bright-green fluorescence emission in the DFA method.

#### 2.4.2. External Parasites

The collected skin scraping samples were used for the morphological characterization and identification of rabbit mites based on the descriptions from Hendrix et al. [21].

#### 2.4.3. Isolation, Identification, and Virulence Characterization of Fungi from Rabbit Fur Samples

Fur samples were inoculated on Sabouraud Dextrose Agar (SDA), as described by Abreu et al. [22]. Following the observation of colony growth, fungal identification was performed based on macroscopical examination of the morphology of the colonies (texture and color) and through microscopic observations of different fungal structures (spores at 1000× total magnification in immersion oil; hyphae and sporangia at 200× and 400× total magnifications) using a lactophenol cotton blue stain and an Olympus BX50 light microscope (Olympus Iberia S.A.U., Barcelona, Spain) [23,24].

Then, mycelium samples were taken from the periphery of each fungal colony to assess the phenotypic expression of five virulence factors: biofilm, DNase, gelatinase, lecithinase, and proteinase production [23,24]. Each mycelium sample was inoculated on different culture media according to each virulence factor and incubated at 25 °C for 72 h. In addition, the gelatinase culture media was then submitted to refrigeration at 4 °C for 30 min. All media and incubation conditions followed the descriptions from Lozano et al. [25] and Raposo et al. [26].

#### 2.4.4. Statistical Analysis

All data from this study was stored and analyzed through descriptive statistics using Microsoft^®^ Excel^®^ (Microsoft Corporation, Redmond, WA, USA, 2024), according to the following epidemiological variables: rabbit origin (internal or external to the hospital), age, outdoor access, district of residence, frequency of housing sanitizing, type of household, and deworming and health status.

Furthermore, data from questionnaires and parasitological analyses were subjected to statistical tests using the software IBM^®^ SPSS^®^ Statistics v27 (IBM Corporation, Armonk, NY, EUA). Associations between parasite frequencies and the tested biotic and abiotic factors were assessed using the Pearson’s Chi-square test or, alternatively, Fisher’s exact test (when the Chi-square test requirements were not fulfilled). A significance level of *p* < 0.05 was used for all tests.

## 3. Results

### 3.1. Characterization of the Study Population

**Figure 1:**
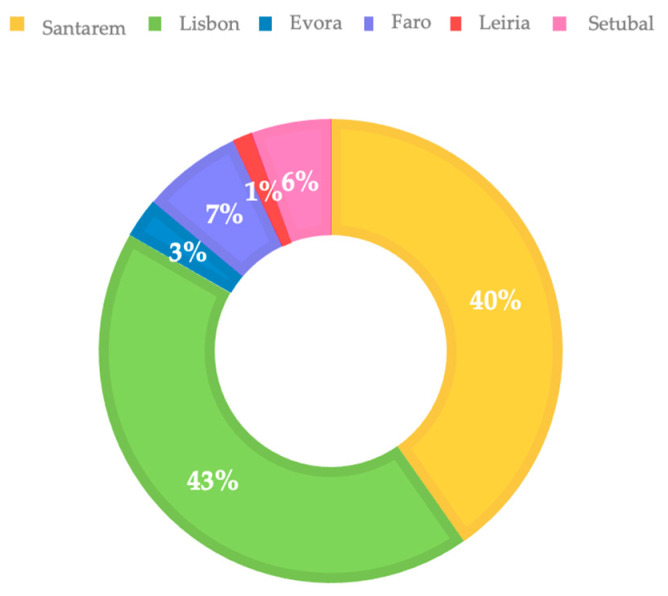
Sampled rabbit distributions according to their district of origin in Portugal: Santarem (yellow color), Lisbon (green), Evora (dark blue), Faro (purple), Leiria (red), and Setubal (pink).

Figure 1 shows the distribution of the rabbits according to their district of origin. Most of the animals included in the study were from Lisbon (43%), followed by Santarém district (40%) (Portugal, mainland).

In a total of 14 households (39%; 14/36), rabbits were cohabitating with immunocompromised individuals: 13 households included children, and 1 had elderly people. From these, the members of six households (43%, 6/14) had contact with animals carrying at least one of the agents tested: three households had rabbits parasitized by nematodes and coccidia; one household had rabbits infected with *Cryptosporidium* spp. and *Giardia* spp.; and two households had rabbits carrying potentially pathogenic fungi.

Among the 69 rabbits tested for the presence of GI parasites, 21 animals (30%) were aged six months or less (thus classified as juveniles), while the remaining were considered as adults (6 months to 14 years-old). Moreover, 58% (40/69) of the rabbits were parasitized with GI parasites. Notably, 95% (20/21) of the juveniles tested positive for GI parasites, compared to 42% (20/48) of the adult rabbits, with these differences being statistically significant (*p* < 0.001).

Regarding housing conditions, 46% of the rabbits were kept in cages, 32% had access to areas external to their primary enclosure, and 53% had either occasional or exclusive access to outdoor areas. Among the rabbits with outdoor access, 85% were infected with GI parasites, in contrast with those kept exclusively indoors and under more controlled conditions, of which only 15% were parasitized. A statistically significant association was found between outdoor access and infection with GI parasites (*p* < 0.001).

As for hygiene practices, the cleaning frequency of the animal’s living environment was as follows: daily in 7% (5/69) of the cases, every two days in 6% (4/69), every three days in 14% (10/69), weekly in 25% (17/69), biweekly in 1% (1/69), monthly in 3% (2/69), and less than once a month in 44% (30/69) of the cases. When correlating the cleaning frequency with the number of GI-parasitized animals, it was determined that most positive cases occurred in environments where cleaning practices were performed less than once per month (*p* < 0.001). Among rabbits housed under these conditions, 30% (9/30) were infected with *Cryptosporidium* spp., *Giardia* spp., or both; 57% (17/30) were infected with coccidia and/or nematodes; and 13% (4/30) were infested with ectoparasites.

Regarding the rabbits’ clinical signs at the sampling timepoints or within the week prior to collection, 12% had diarrhea, although only 3% of these rabbits tested positive for GI parasites.

As for internal deworming, 23% (16/69) of the sampled animals were dewormed within the previous six months, whereas the remaining were either not dewormed or had their antiparasitic treatments outdated. In all dewormed animals, fenbendazole was the antiparasitic molecule of choice (100%), and only two of these rabbits (13%, 2/16) presented GI parasites. In contrast, most of the rabbits not subjected to deworming protocols (72%; 38/53) were parasitized. A statistically significant association was observed between GI parasitological results and the deworming status of the animals (*p* < 0.001). Within the studied rabbit population, 60% had contact with other animals, and of which, only five (12%; 5/43) had been previously dewormed.

### 3.2. Results for GI Parasitism

**Figure 2:**
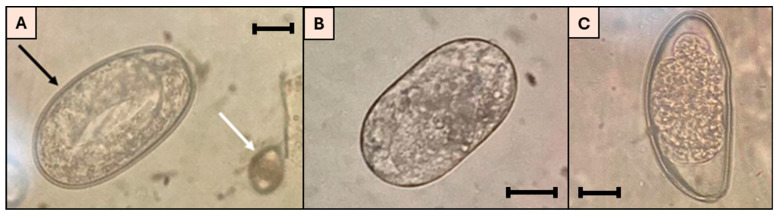
(**A**) *G. strigosum* egg (black arrow) and coccidia oocysts (white arrow); (**B**) *T. retortaeformis* egg; (**C**) *P. ambiguus* egg; scale bars: 20 µm (original photos).

A total of 69 rabbits were tested for the presence of GI parasites, of which 40 (58%) tested positive for these agents. Among the parasitized rabbits, 15 (38%) were infected with nematodes, including *G. strigosum*, *T. retortaeformis*, and *P. ambiguus* (Figure 2).

Moreover, 13% of the rabbits (9/69) were infected with *G. strigosum*. Of these, six animals exhibited a parasitic shedding within the range of (0–10) EPG, two within the range of (10–20) EPG, and only one in the range of (20–30) EPG. Considering the nematode *T. retortaeformis*, 17% of the rabbits (12/69) were diagnosed as positive. Of these, seven animals exhibited parasitic shedding within the range of (0–250) EPG, two rabbits had a shedding between (250–500) EPG, and one rabbit showed an egg count within the range of (500–750) EPG. Among the two rabbits with the highest parasitic shedding, one presented an EPG count in the range of (1000–1250) and the other in the range of (1500–1750) EPG. The nematode *P. ambiguus* showed a frequency of 13% (9/69), with eight animals exhibiting a parasitic shedding within the range of (0–100) EPG, while one rabbit had an EPG count in the range of (300–400).

**Figure 3:**
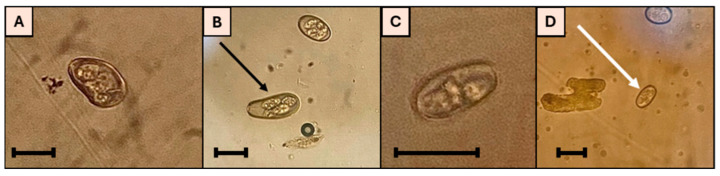
(**A**) Oocyst of *E. perforans* (25 × 12 µm, elliptic shape); (**B**) oocyst of *E. magna* (51 × 22 µm, elliptic shape, black arrow); (**C**) oocyst of *E. piriformis* (29 × 20 µm, pyriform shape); (**D**) oocyst of *E. exigua* (21 × 10 µm, elliptic shape, white arrow); scale bars: 20 µm (original photos).

Regarding coccidia infections, four species of *Eimeria* were identified in the rabbits’ fecal samples: *Eimeria exigua*, *E. piriformis*, *E. magna*, and *E. perforans* (Figure 3).

A total of 31 rabbits (45%, 31/69) were positive for coccidia oocysts, all belonging to the genus *Eimeria*. In these animals, nineteen had a parasitic shedding within the range of (0–15.000) OPG, one rabbit had an OPG count in the range of (15.000–30.000), five rabbits exhibited a coccidia shedding in the range of (30.000–45.000) OPG, four rabbits had OPG counts within the range of (45.000–60.000), and two rabbits had the highest coccidia shedding counts, within the range of (60.000–75.000) OPG. The lowest coccidia shedding among the positive rabbits was 20 OPG, with 25% percent of the animals having an oocyst shedding lower than 520 OPG. The median parasitic shedding reached 10.080 OPG, with 75% of cases lower than 35.680 OPG. Finally, the highest shedding value totalized 65.960 OPG.

**Figure 4:**
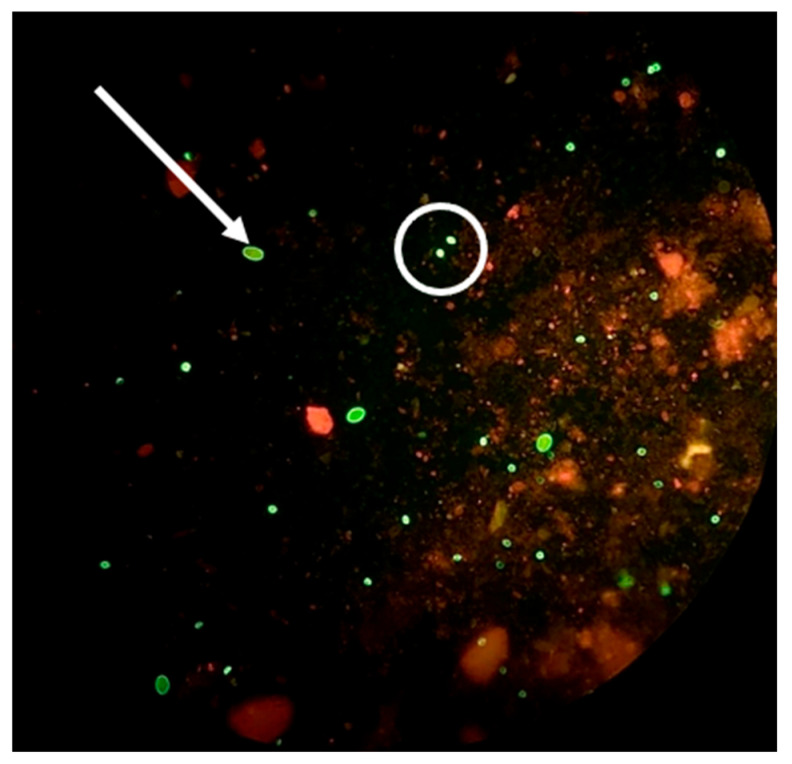
*Giardia* sp. cyst (white arrow) and a *Cryptosporidium* sp. oocyst (white circle) identified using the DFA technique (original photo).

Regarding the results obtained with the DFA tests, it was found that 32% (22/69) of the fecal samples were positive for *Cryptosporidium* spp. oocysts, and 9% (6/69) were positive for both *Cryptosporidium* spp. oocysts and *Giardia* spp. cysts (Figure 4). However, the same was not observed using the MZN technique, with no positive cases being found for these protozoan species.

### 3.3. Results for Ectoparasite Infestation

**Figure 5:**
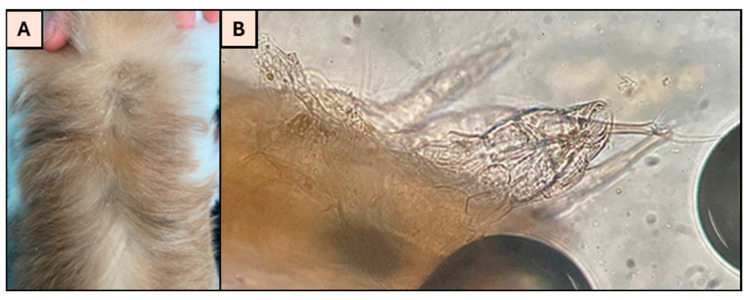
(**A**) Skin desquamation due to *Cheyletiella* spp. infestation; (**B**) *Cheyletiella* sp. and its pedicels, examined at 400× total magnification, using an Olympus BX50 light microscope (original photos).

A total of 10% (7/72) of the animals tested positive for mite infestation, all identified as *Cheyletiella* spp. (Figure 5). Moreover, 47% of the rabbits showed signs of pruritus, and a statistically significant association was found between pruritus and the presence of ectoparasites (*p* < 0.005). It was also noted that 85% (n = 6) of the rabbits with ectoparasites also had GI parasites, such as nematodes and/or coccidia.

### 3.4. Fungi Isolation and Assessment of Their Virulence Profile

A total of eight rabbits (11%; 8/72) exhibited lesions compatible with fungal infection. Of these, five rabbits were found to be carriers of *Penicillium* spp., *Rhizopus* spp., or *Scopulariopsis* spp., and it was possible to obtain a total of two *Penicillium* spp. isolates (P1 and P2), four *Rhizopus* spp. isolates (R1, R2, R3, and R4), and one *Scopulariopsis* sp. isolate (S1).

**Figure 6:**
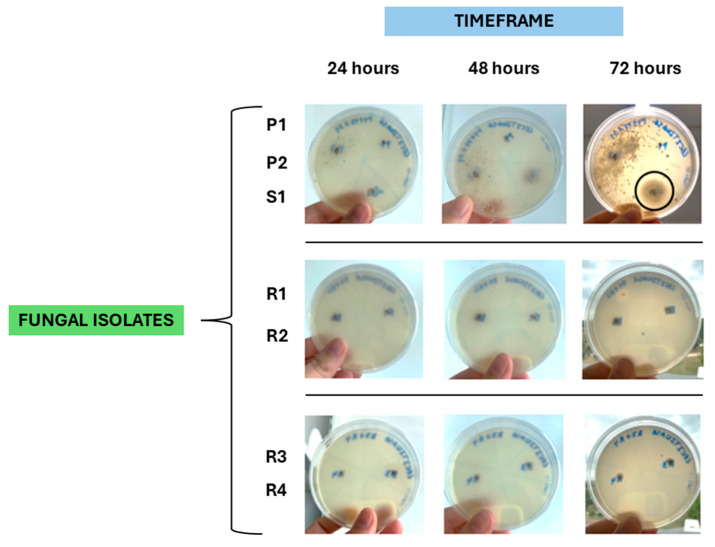
Assessment of lecithinase expressions by two isolates of *Penicillium* sp. (P1 and P2), one isolate of *Scopulariopsis* sp. (S1), and four isolates of *Rhizopus* sp. (R1, R2, R3, and R4); a positive result was registered for isolate S1 after 72 h of incubation (black circle) (original photos).

**Figure 7:**
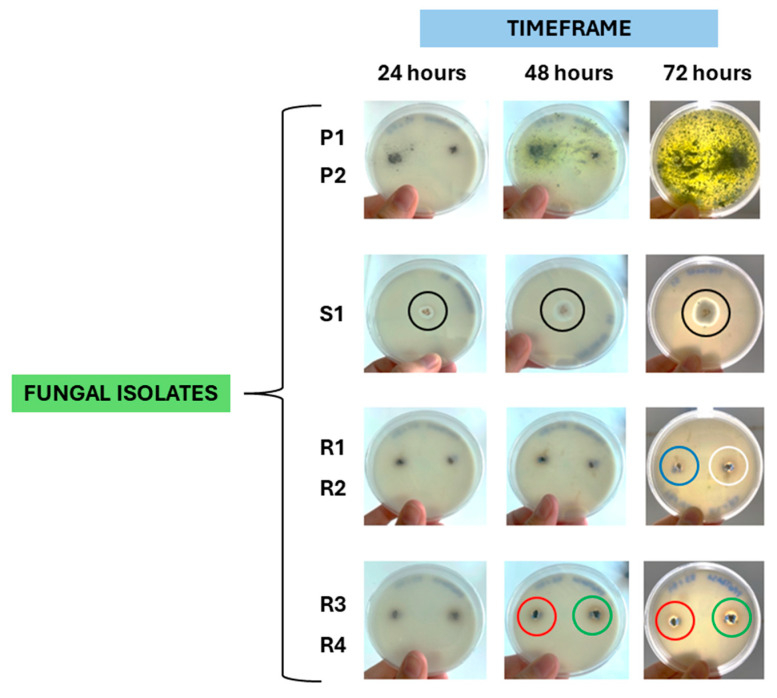
Assessment of proteinase expressions by two *Penicillium* spp. isolates (P1, P2), one *Scopulariopsis* sp. isolate (S1), and four *Rhizopus* spp. isolates (R1, R2, R3, R4). Isolate S1 tested positive after 24 h (black circle), isolates R1 (blue circle) and R2 (white circle) after at 72 h, and isolates R3 (green circle) and R4 (red circle) after 48 h of incubation (original photos).

**Figure 8:**
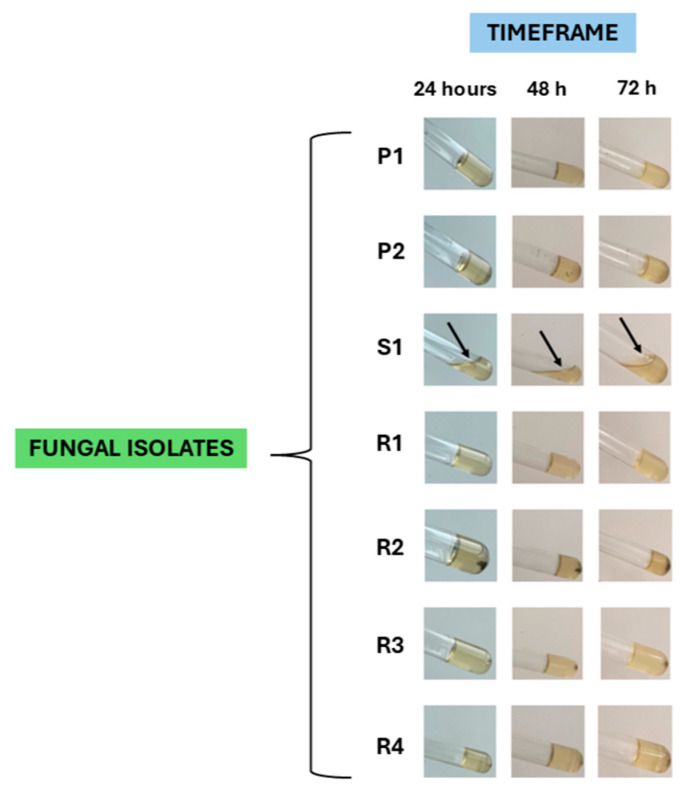
Assessment of gelatinase productions by two *Penicillium* spp. isolates (P1, P2), one *Scopulariopsis* sp. isolate (S1), and four *Rhizopus* spp. isolates (R1, R2, R3, R4); a positive result was recorded for isolate S1 at 24 h (black arrow); all images were taken after incubation at 4 °C for 30 min (original photos).

Regarding the virulence profile of these isolates, it was found that the *Scopulariopsis* sp. isolate S1 tested positive for lecithinase activity after 72 h of incubation (Figure 6); five isolates were able to produce proteinase, namely *Scopulariopsis* sp. isolate S1 (after 24 h of incubation), *Rhizopus* spp. isolates R1 and R2 (after 72 h of incubation), and *Rhizopus* spp. isolates R3 and R4 (after 48 h of incubation) (Figure 7); the *Scopulariopsis* sp. isolate S1 was able to express gelatinase after 24 h (Figure 8); and none of the fungal isolates were able to express DNase and produce biofilms.

Overall, the *Scopulariopsis* sp. isolate S1 tested positive for 60% of the virulence factors, the four *Rhizopus* spp. isolates (R1, R2, R3, and R4) were positive for 20% of the virulence factors, while both *Penicillium* spp. isolates (P1 and P2) tested negative for all virulence factors.

## 4. Discussion

This study assessed the presence of GI parasites, ectoparasites, and potentially pathogenic fungi in rabbits attending consultation at the HEV-FMV-ULisboa and from other sources. The origin of the rabbits, the characteristics of each animal, and the exposure conditions were evaluated to better understand the influence of each biotic and abiotic factor in the presence or absence of the studied pathogens.

It was found that coccidia were the most frequent parasites (45%) in the studied rabbit population, followed by the nematodes *T. retortaeformis* (17%), *P. ambiguus* (13%), and *G. strigosum* (13%). Recent screening studies in pet rabbits attending consultation in Portugal registered a lower frequency of *Eimeria* spp. (12–13%) [2,3]. The higher frequencies in our study may result from inadequate hygiene practices, which enable the establishment of increased environmental concentrations of infectious sporulated oocysts. In future studies, it would also be important to assess the specific cleaning protocols applied to more accurately evaluate their role in parasite frequency and shedding. Previous research in exotic mammals conducted at HEV-FMV-ULisboa identified a lower prevalence for *P. ambiguus* (3%), with it being the only GI parasite identified, which could be explained by the lower number of samples collected and the high percentage of rabbits that had been dewormed (70%) [5].

The implementation of the Mini-FLOTAC technique allowed for the detection of a wide range of coccidia oocyst shedding, from 20 OPG to 65.960 OPG, averaging 32.990 OPG. Comparing the positive results for coccidia with the health status of the rabbits, it was found that only 12% of the animals were clinically ill. In this study, it was possible to identify four species of *Eimeria* spp.—*E. magna*, *E. piriformis*, *E. exigua*, and *E. perforans*—which are often reported in the literature as having moderate-to-low pathogenicity, thus corroborating our findings, with a low proportion of parasitized animals showing clinical signs [27]. The findings reported by Szkucik et al. [28] were consistent with ours, mentioning that clinical signs, like acute diarrhea or even mortality, can occur particularly when highly pathogenic species such as *E. flavescens* and *E. intestinalis* are present. In future studies, it would be interesting to combine morphological characterization with molecular methods, using, for example, the single oocyst selection technique [29].

In our study, young rabbits were more parasitized with *Eimeria* spp. when compared to adults. This finding corroborates a previous study in farmed rabbits, which stated that a rabbit’s age appears to have a significant impact on the degree of *Eimeria* spp. infection, indicating a higher prevalence in rabbits under six months. Additionally, it also observed that coccidia predominate in rabbits aged between five and six weeks, immediately after weaning, when their immature immune system turns them more susceptible to infectious agents [30]. Similarly, a study performed in pet rabbits in Finland estimated that the probability of being parasitized with *Eimeria* spp. was 6.5 times higher in young rabbits and 5.2 times higher in rabbits housed outdoors [31].

With the DFA technique, 32% of the examined samples tested positive for *Cryptosporidium* spp. and 9% were positive for *Giardia* spp., while MZN did not detect any positive case for both these protozoan agents, thus reflecting a higher sensitivity of analysis for the DFA technique, which is in accordance with the existent literature for other animal host species, namely ruminants and reptiles [19,32]. Among the households examined, one included rabbits infected with these potential zoonotic agents, thereby underscoring the necessity of providing a diagnosis and treatment of the animals [31]. In fact, the zoonotic potential of *C. cuniculus* was already described in a diarrhea outbreak in humans, as reported in the United Kingdom [9]. However, a recent study also evaluated the zoonotic risk for *C. cuniculus* and mentioned that despite its abundance in rabbits, reports in humans are relatively rare and have declined substantially in the UK. This species seems to be largely host-specific, and thus, its zoonotic risk is relatively low [33].

Regarding deworming practices, it was observed that 13% of rabbits that were correctly dewormed with fenbendazole presented GI parasites, while 72% of non-dewormed rabbits were parasitized, with these differences being significant (*p* < 0.001). A study performed in Finland reported that 77.6% of anthelminthic treatments in pet rabbits included a fenbendazole formulation, 20.4% flubendazole, and 2% pyrantel pamoate [31]. These authors also mentioned that pellets containing coccidiostats, like robenidine, were used to reduce clinical signs and oocyst excretion, being freely available in Finnish pet stores, which might lead to its misuse and, consequently, to an increase in the problem of drug resistance. A recent study also reported that prolonged anticoccidial drug use could lead to coccidia resistance and drug residues in food; therefore, the development of recombinant vaccines (namely using recombinant proteins for highly pathogenic species of *E. intestinalis*) is required to control rabbit coccidiosis [34]. Another study considered surface antigens (SAGs), which are involved in the invasion of coccidia into the host, to be potential antigens to be explored as targets for vaccination [35].

Our study found that hygiene practices frequency had a noteworthy impact on GI parasitism in rabbits, with a significant association being recorded between hygiene intervals less than one month and exceeding one month and the presence of *Cryptosporidium* spp., *Giardia* spp., coccidia, and nematodes. Previous studies also emphasized the importance of housing hygiene practices in preventing intestinal diseases in rabbits [36]. In fact, a study on the health and welfare of farmed rabbits mentioned that GI, dermatological, and reproductive conditions and neonatal morbidity can be reduced by securing appropriate biosafety measures, such as cleaning the housing equipment, removing feces, and controlling insects and rodents [37].

A total of seven rabbits (10%) were infested with mites of the *Cheyletiella* genus. The mite’s taxon and its prevalence emphasized the need to proceed with its screening and treatment, particularly in the presence of pruritus. Similarly, a previous study conducted at the same veterinary hospital described a prevalence of 10.81% for *C. parasitovorax*; however, a higher prevalence of mites was identified (21.62%) (8.11% *L. gibbus* and 2.70% *P. cuniculi*) [5]. Also, a higher prevalence (38%) was also reported in a study performed in 50 farm rabbits in Iran, with authors highlighting that results were due to poor sanitary conditions and lack of external deworming [38].

Pet rabbits can be potential vehicles for zoonotic or allergenic fungi to their owners. In this study, environmental saprophytic filamentous fungi, including *Penicillium* spp., *Rhizopus* spp., and *Scopulariopsis* spp., were isolated and assessed for the phenotypic expression of lecithinase, DNase, proteinase, biofilm, and gelatinase. The most relevant results were found for the *Scopulariopsis* sp. isolate S1, which tested positive for 60% of the virulence factors—proteinase, lecithinase, and gelatinase. This finding aligns with the report from Abreu et al. [22], stating that after abrasive or perforating injury, the *Scopulariopsis* genus may have the ability to cause disease not only in immunocompromised or nutritionally deficient animals but also in healthy ones [22]. Similarly, Bourguet et al. [39] considered that *Scopulariopsis brevicaulis*, the most frequently *Scopulariopsis* species found in mammals, is occasionally pathogenic, potentially causing skin or ocular lesions [39]. Studies also emphasize the importance of rabbit grooming as a natural hygiene practice, with its absence increasing the risk of acquiring skin lesions, like dermatomycosis [22,40].

A limitation of the present study is the relatively small number of animals sampled 11% (8/72), particularly their fur samples, which restricts the ability to generalize these findings to the wider rabbit population in Portugal. Nevertheless, these findings highlight the importance of considering environmental fungi in the differential diagnosis of skin diseases, besides dermatophytes, and assessing their potential zoonotic risk. In this study, samples were collected only from animals showing clinical signs of skin disease, but future studies could also be performed in asymptomatic animals to establish a link between the presence of these potentially virulent fungi in rabbits without skin disease, aiming at better understanding their clinical impact. Including the sampling of asymptomatic rabbits would likely provide valuable data regarding the prevalence of environmental and commensal fungi and a more accurate estimation of the presence of fungi in rabbit skin. Additionally, it could reveal the presence of potential zoonotic agents in apparently healthy rabbits without active skin lesions, thereby redefining the risk assessment for owners and advising them for more effective preventive measures.

Finally, further large-scale studies could include the assessment of potential sources of infection for these pathogens (e.g., water, food, environment) and associations with other potential biotic and abiotic risk factors, like climate, sex, type of nutrition, interactions between GI and ectoparasites, and others, to better understand the transmission and pathogenicity of GI and ectoparasites and environmental fungi in rabbit collections. Also, addressing anthelmintic resistance and testing the effectiveness of different drugs against several GI and ectoparasites would be highly useful to perform in further studies in rabbits. Moreover, the search for other agents with zoonotic potential, like the microsporidian parasites *Enterocytozoon bieneusi*, *Encephalitozoon cuniculi*, and *Encephalitozoon intestinalis*, and performing molecular diagnosis in fecal and blood samples in rabbits attending veterinary clinics could add noteworthy information concerning the distribution of these parasitic and fungal agents in rabbit populations and help establish better prevention and treatment protocols within a One Health framework

## 5. Conclusions

To the authors best knowledge, this was the first study in Portugal to target the diagnosis of GI and external parasite infections/infestations in rabbits and to simultaneously assess their fur mycobiota during clinical consultations or on samples from private owners or breeders. Protozoans of the genus *Eimeria* represented the most frequently diagnosed GI parasites in juvenile rabbits, followed by *Cryptosporidium* spp. and *Giardia* spp., whereas ectoparasitism was only caused by mites of the genus *Cheyletiella*. Also, two *Penicillium* isolates, four *Rhizopus* isolates, and one *Scopulariopsis* isolate were obtained from these rabbits’ furs, with the last isolate testing positive for the expression of proteinase, lecithinase, and gelatinase activities. Overall, the results from this study suggest the implementation of Mini-FLOTAC and DFA in the routine diagnosis of GI parasitic infections and the isolation of fungi and assessment of their virulence profile as complementary diagnostic examinations in rabbit medicine within a One Health framework.

## Data Availability

The raw data supporting the conclusions of this article will be made available by the authors on request.

## Supplementary Materials

The following supporting information can be downloaded at: https://www.mdpi.com/article/10.3390/microorganisms13092146/s1, Supplementary File S1: Informed consent and questionnaire to rabbit owners.

## Abbreviations

The following abbreviations are used in this manuscript:

CIISA-FMV-ULisboa	Centre for Interdisciplinary Research in Animal Health
DFA	Direct Immunofluorescence Antibody
DTM	Dermatophyte Test Medium
EPG	Eggs Per Gram
GI	Gastrointestinal
HEV-FMV-ULisboa	Hospital Escolar Veterinário da Faculdade de Medicina Veterinária, Universidade de Lisboa
LPDP	Laboratory of Parasitology and Parasitic Diseases
MZN	Modified Ziehl–Neelsen
OPG	Oocysts Per Gram
SDA	Sabouraud Dextrose Agar
