# Supplementary material for: Gastrointestinal Parasites, Ectoparasites, and Fungi in Rabbits Attending Clinical Consultations and from Private Owners and Breeders in Portugal
Source: Microorganisms. 2025 Sep 13;13(9):2146. doi: 10.3390/microorganisms13092146 (PMC12472197; doi:10.3390/microorganisms13092146)
Supplement: Supplementary file 1 [file microorganisms-13-02146-s001.zip › microorganisms-3814981-supplementary.pdf]

Supplementary file S1 – "Informed consent and questionnaire to rabbits' owners"

1) Informed consent

I, ___________________________________, signed below, holder of the Portuguese Citizen Card / Passport ________________ hereby authorize Carolina Silvano Valadão do Vale, from the Faculty of Veterinary Medicine – University of Lisbon, to perform samplings on my animal, named__________________, in the scope of her research project within the Integrated Master Thesis work entitled: "Searching for gastrointestinal parasites, ectoparasites and fungi with pathogenic potential in rabbits". I understood that the presence of endoparasites is often not diagnosed during clinical consultations, and that the absence of clinic signs does not mean necessarily a negative diagnosis for the presence of these agents. Younger animals or those subjected to improper management conditions (nutrition and environment), stress or concomitant diseases, have a higher susceptibility of developing disease associated to these agents, which often lead to prostration, loss of appetite and weight, growth delay, diarrhea, and even death. Thus, its screening is of upmost importance. Control can be achieved through proper treatment, adapted to each parasite, and by means of improving management conditions. The control of ectoparasites is performed through direct observation of the entire fur in the physical exam, which can be complemented with other diagnostic procedures, namely trichogram, superficial and deep scratching, and using adhesive-tape. These techniques are important for a comprehensive diagnosis of infections from these parasites, which often pose serious risks for animal welfare, and eventually lead to prostration, itching, hyperkeratosis, excoriation, alopecia, anemia, and even secondary infections. I was informed that samplings are free of charge, and non-invasive to the animal. I'm hereby declaring that I was informed, both verbally and written, about the conditions in which this research project is being developed, and that I'm free to remove the above-mentioned patient from the current project at any time. In the scope of the General Regulation for Personal Data Protection (Regulation 2016/679), the code of conduct for using non-human animals, the European Union Directives (Directive 2010/63/EU) related to the protection of animals used in scientific research, Regulation (EU) 2019/1010 from 5 June, and the Commission Decision 2020/569/EU, I'm hereby authorizing the use of all data, clinical exams and digital contents from the respective animal for the publication of Integrated Master Dissertations, Doctoral Thesis, and any other scientific studies.

Lisbon, (date)

The Owner,

(signature)

2) Questionnaire to rabbits' owners

Master Dissertation Project

"Searching for gastrointestinal parasites, ectoparasites and fungi with pathogenic potential in rabbits"

Questionnaires to the owners

By answering this questionnaire, you are contributing to the advancement of parasitology in exotic animals.

Your contribution is of upmost importance!

Your personal data is confidential and will only be used for this study.

I. Owner information

Name: ______________________

Address (Parish / Municipality of Residence): ______________

Household information:

- ☐ Children (<12 years)
- ☐ Elderly (>65 years)
- ☐ Other (disease/disability)

II. Companion animal information

**Name:** ____________	**If yes, how many?**
**Age:** ____	☐ 1
**Sex:**	☐ 2
☐ F	☐ >2. **How many?** ___
☐ M	**If yes, which animals?**
**Animal nr.** ________	☐ Rabbit
**Contact with other animals:**	☐ Dog
☐ Yes	☐ Cat
☐ No	☐ Others: ____________
☐ Don't know	____________________

Housing:

- ☐ Cage
- ☐ Park
- ☐ Other: ________

Access to areas outside the housing:

- ☐ Yes
- ☐ No

Outdoor access (backyard, balcony):

- ☐ Yes
- ☐ No

If yes, with what frequency:

- ☐ 1-2x per week
- ☐ 1x per day
- ☐ Always
- ☐ Other: ________

Housing sanitizing frequency:

- ☐ Daily
- ☐ 2-2 days
- ☐ 3-3 days
- ☐ Weekly
- ☐ 2-2 weeks
- ☐ Other: ________

Interaction frequency with the animal:

- ☐ We do not interact
- ☐ <1 hour daily
- ☐ <2 hours daily
- ☐ >3 hours daily

Previously diagnosed diseases/comorbidities:

____

____

____

____

Having or had diarrhoea?

- ☐ Yes
- ☐ No
- ☐ Don't know

If yes, of what type?

- ☐ With blood
- ☐ Yellowish
- ☐ Brownish
- ☐ Liquid
- ☐ Mucous
- ☐ Pasty
- ☐ Other: ________

Any previous coprology?

- ☐ Yes. When? ______
- ☐ No
- ☐ Don't know

If yes, how was the result?

- ☐ Positive
- ☐ Negative
- ☐ Unconclusive
- ☐ Don't know

If yes, was he/she treated?

- ☐ Yes
- ☐ No
- ☐ Don't know

Having or had any itching?	Any previous ectoparasite screening?
☐ Yes	☐ Yes. When? ______
☐ No	☐ No
☐ Don't know	☐ Don't know
If yes, with what frequency?	If yes, how was the result?
☐ Highly frequent	☐ Positive
☐ Frequently	☐ Negative
☐ Sometimes	☐ Unconclusive
☐ Rarely	☐ Don't know
☐ Never	If yes, was he/she treated?
If yes, where?	☐ Yes
☐ Ears	☐ No
☐ Fingers / interfinger areas	☐ Don't know
☐ Back	
☐ Entire body	
☐ Other: ____________	

III. Information about companion animal's deworming

Do he/she get dewormed?	Deworming frequency:
☐ Yes	☐ Monthly
☐ No	☐ 2-2 months
☐ Don't know	☐ 3-3 months
	☐ 6-6 months
If yes, with what dewormer(s)?	☐ Annually
____________	☐ Other: ________
____________	In case of having other animals, are they dewormed?
____________	☐ Yes
If yes, when was the previous deworming?	☐ No
____________	☐ Don't know
	☐ Not applied

IV. Questions and comments:

____

____

____

____
